# Coat of many colours—DNA reveals polymorphism of mantle patterns and colouration in Caribbean *Cyphoma* Röding, 1798 (Gastropoda, Ovulidae)

**DOI:** 10.7717/peerj.3018

**Published:** 2017-03-02

**Authors:** Bastian T. Reijnen, Sancia E.T. van der Meij

**Affiliations:** 1 Naturalis Biodiversity Center, Leiden, The Netherlands; 2 Oxford University Museum of Natural History, University of Oxford, Oxford, United Kingdom; 3 Linacre College, Oxford, United Kingdom

**Keywords:** Gastropoda, Marine invertebrates, Molecular phylogeny, Octocorallia, Taxonomy, Systematics

## Abstract

The iconic gastropod genus *Cyphoma* is commonly observed in the Caribbean, where it lives in association with various octocorallian hosts. Each species in the genus *Cyphoma* has a unique, characteristic mantle pattern and colouration, which separates the valid taxa. Because of its abundance and recognisability *Cyphoma gibbosum* has been used as a model organism in several studies concerning allelochemicals, reef degradation, and physical defence mechanisms. Molecular analyses based on four molecular markers (COI, 16S, H3 and 28S) for three *Cyphoma* species (*C. gibbosum*, *C. mcgintyi*, *C. signatum*) and an unidentified black morph, collected from three localities in the Caribbean, show that they represent morphological varieties of a single, genetically homogeneous species. This outcome is in agreement with previous anatomical studies. As a result *C. mcgintyi* and *C. signatum* are synonymised with *C. gibbosum*, which is a key result for future work using *C. gibbosum* as a model organism. The striking morphological differences in mantle pattern and colouration are hypothesised to be the result of one of three possible scenarios: rapid divergence, supergenes (including balanced polymorphism), or incipient speciation.

## Introduction

Biodiversity on reefs is dominated by highly diverse invertebrate taxa that are understudied and incompletely described (Reaka-Kudla, 1997). Many of these taxa live in association with corals on which they rely for food, habitat and settlement cues. Arthropods are the most numerous associated taxa on stony corals, followed by molluscs (Stella et al., 2011). For Octocorallia no such review on their associated fauna is available, but Goh, Ng & Chou (1999) reported on 30 mollusc species among 17 families living in association with gorgonians in Singapore. This gorgonian associated fauna included bivalves (e.g., *Pteria*), nudibranchs (e.g., *Phyllodesmium*, *Tritonia*), and gastropods (Ovulidae). The widespread family Ovulidae occurs in all temperate and tropical oceans and all but one species, *Volva volva* (Linnaeus, 1758), live intrinsically associated with Octocorallia and Antipatharia (Cate, 1973; Lorenz & Fehse, 2009). Ovulids roam the branches of their host corals and feed on the polyps and tissue (Gerhart, 1990). Ovulids have a mantle, which can cover their entire shell; the different colours, patterns and appendices provide camouflage or conversely advertise their toxicity with conspicuous, aposematic mantle patterns and colourations (Rosenberg, 1992).

The well-known Atlantic ovulid species *Cyphoma gibbosum* (Linnaeus, 1758) is a conspicuous and easily recognisable species living on various soft coral and gorgonian species throughout the Caribbean (Simone, 2004; Lorenz & Fehse, 2009; Reijnen, Hoeksema & Gittenberger, 2010; Humann, DeLoach & Wilk, 2013) and can locally occur in high densities (Chiappone et al., 2003). It is therefore often used as a model organism and has been used in studies dealing with allelochemicals and physical defence systems (Van Alstyne & Paul, 1992; Vrolijk & Targett, 1992; Whalen et al., 2010), studies on their association with fungal diseases in Caribbean gorgonians (Rypien & Baker, 2009) and research on reef degradation and predation (Gerhart, 1990; Burkepile & Hay, 2007; Evans, Coffroth & Lasker, 2013). The genus *Cyphoma* has 14 extant species recognised by Lorenz & Fehse (2009) and 13–15 extant species according to Rosenberg (2015). Two *Cyphoma* species are not found in the Atlantic Ocean but instead have an East Pacific distribution, namely *C. emarginata* (Sowerby I, 1830) and *C. arturi* Fehse, 2006. All other *Cyphoma* occur in the Atlantic on shallow reefs (intertidal) and in deep water (1,200 m), from Florida to southern Brazil, and from the Caribbean to the Canary Islands and St. Helena (Lorenz & Fehse, 2009; Humann, DeLoach & Wilk, 2013). The genus is assumed to be monophyletic, and is part of the subfamily Simniinae (Schiaparelli et al., 2005; Fehse, 2007). Apart from *C. gibbosum,* most *Cyphoma* species are relatively rare (Lorenz & Fehse, 2009) and as a result there are fewer studies on other *Cyphoma* species. Botero (1990), Ruesink & Harvell (1990), and Reijnen, Hoeksema & Gittenberger (2010) studied the host species of *C. signatum*, whereas Ghiselin & Wislon (1966) studied the anatomy, natural history and reproduction of this species. Recently two new host records (*Plexaurella grandiflora* Verrill, 1912 and *Muriceopsis sulphurea* (Donovan, 1825)) were published for *C. macumba* Petuch, 1979 observed in northeastern Brazil (Pinto, Benevides & Sampaio, 2016). Apart from the aforementioned studies there are no records of *Cyphoma* species, other than *C. gibbosum*, in the scientific literature.

**Figure 1:**
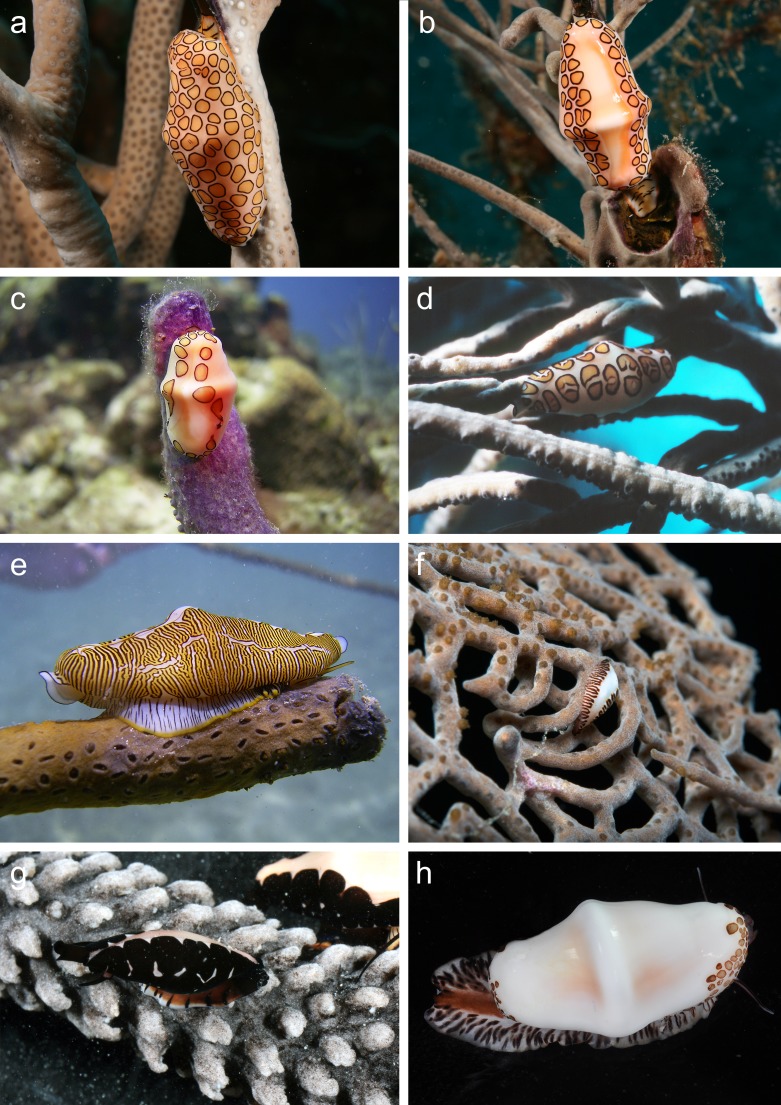
*In situ* photographs of *Cyphoma* species. *Cyphoma* species showing different mantle patterns and colouration. (A) *Cyphoma gibbosum* on *Pseudoplexaura* sp. (B) *C. gibbosum* on *Pseudoplexaura* sp. (C) *C. gibbosum* with atypical mantle pattern (only dots around mantle edges) on *Briareum asbestinum* (D) *C*. cf. *allenae* on *Antillogorgia americana* (E) *C. signatum* on *Plexaurella dichotoma* (see Reijnen, Hoeksema & Gittenberger, 2010: Fig. 1B) (F) Juvenile *C. signatum* on *Gorgonia ventalina* (G) *Cyphoma* “black morph” on *Eunicea tourneforti* (H) *C. mcgintyi* from Florida, USA. Photos: (A–G) B.T. Reijnen, all from Curaçao; (H) Florida Museum of Natural History.

**Figure 2:**
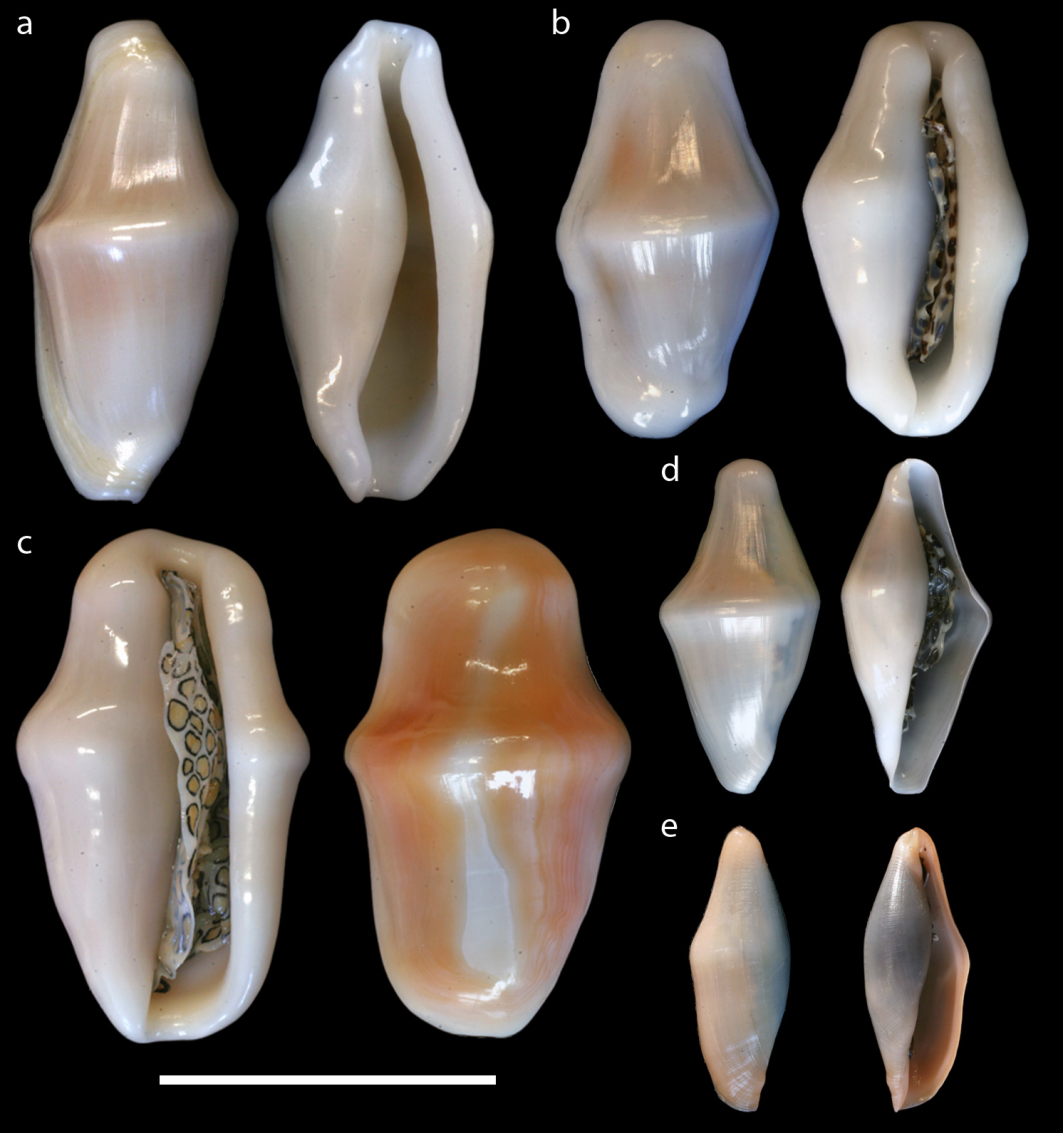
Dorsal and ventral views of *Cyphoma* shells. Dorsal and ventral views of the shells of *Cyphoma* species in this study. (A) *Cyphoma signatum* (RMNH.Mol.100828) (B) *C. mcgintyi* (UF.446893a) (C) *C. gibbosum* (UF.446879) (D) *C. mcgintyi* (UF.446893b; juvenile) (E) *Cyphoma* “black morph” (RMNH.Mol.337800).

The majority of *Cyphoma* species can be identified with the help of characteristic patterns and colouration of their mantle, which are considered species specific in Ovulidae (Cate, 1973; Mase, 1989; Fig. 1). There are, however, observations of mantles showing intermediate patterns (e.g., Lorenz & Fehse, 2009: A197). In the 18th and 19th century soft tissue, including the mantle, was often not available or recorded and therefore minor shell morphological features (e.g., more pronounced keel, slightly more dentate labrum etc.) were used to separate species (Röding, 1798; Dall, 1897). The species described during this period were later synonymised with *Cyphoma gibbosum*, *C. signatum* and *C. mcgintyi* (see Lorenz & Fehse, 2009). Based on shell morphology alone these three species are also difficult to identify. For example, *C. signatum* and *C. mcgintyi* are differentiated from *C. gibbosum* based on their respective colour patterns (fingerprint pattern vs. brown dots), but based on just shell morphological features *C. signatum* and *C. mcgintyi* can hardly be distinguished (Cate, 1973). Shell morphological features can be used to separate *C. gibbosum* and *C. signatum* by using the differences in shell outline (oval vs. rhomboid) and shell colour (often orange in *C. gibbosum*; Fig. 2). Interpretation of the anatomical features in *Cyphoma*, such as penis form and the size of the osphradium leaflets, are troublesome and no clear differences between species are observed (Ghiselin & Wislon, 1966; Simone, 2004).

Besides the typical species-specific mantle patterns some uncommon *Cyphoma* morphotypes have been recorded (e.g., Lorenz & Fehse, 2009: A202–204; Humann, DeLoach & Wilk, 2013: p. 175). Because of their unusual appearance and apparent rarity, these morphotypes have not yet been identified to species level, or formally described as separate species, and their status remains uncertain.

To investigate the genetics behind the morphological differences in shell shape, mantle patterns and colouration in *Cyphoma* spp. more closely, we used data obtained for a previous study on *Cyphoma* (Reijnen, Hoeksema & Gittenberger, 2010) and supplemented that dataset with an additional 26 specimens belonging to three ovulid species and one unidentified morphotype, and with two additional markers. Here we show the results of phylogenetic analyses based on four molecular markers (COI mtDNA, 16S mtDNA, 28S tDNA and H3 nDNA) for three valid *Cyphoma* species and one unidentified black morph (Fig. 1G), as well as three temperate Atlantic representatives of the subfamily Simniinae (*Cymbovula acicularis* (Lamarck, 1810), *Neosimnia spelta* (Linnaeus, 1758) and *Simnia patula* (Pennant, 1777)).

## Material and Methods

### Collecting

**Figure 3:**
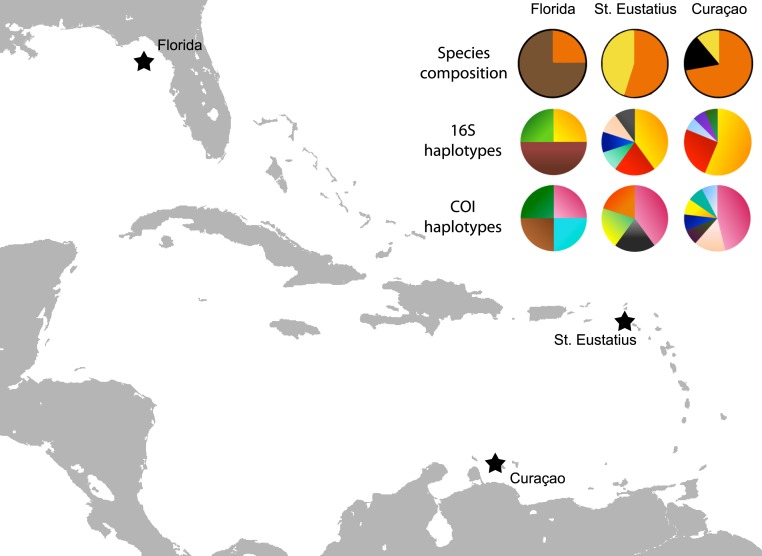
Map with localities. Localities from which the *Cyphoma* spp. and other ovulids were collected in the Caribbean. The species composition per locality is provided (orange, *C. gibbosum*; yellow, *C. signatum*; brown, *C. mcgintyi*; black, *Cyphoma* “black morph”) as well as haplotype frequencies for 16S and COI.

*Cyphoma* specimens and their host corals were collected during fieldwork on the leeward side of Curaçao in 2005 and 2013, and from St. Eustatius in 2015 (Fig. 3). Research on Curaçao was performed under the annual research permit (48584) issued by the Curaçaoan Ministry of Health, Environment and Nature (GMN) to the CARMABI foundation. The valid *Cyphoma* species co-occurred at the sample localities. When possible *in situ* photographs were made to document the mantle patterns and colouration. Subsamples were taken from the host corals for their identification based on sclerite morphology. All specimens were preserved in 80% ethanol and deposited in the mollusc and coelenterate collection of Naturalis Biodiversity Center, Leiden, The Netherlands (collection coded as RMNH.Mol and RMNH.Coel). Three samples of *Cyphoma mcgintyi* and one additional sample of *C. gibbosum*, collected in Florida, were obtained from the Florida Museum of Natural History (FLMH; Table S1). Identification of the snails was based on Kaicher (1991), Fehse (2003), Lorenz & Fehse (2009) and Humann, DeLoach & Wilk (2013), the octocoral hosts were identified with the help of Bayer (1961).

The earlier identification of *Simnialena uniplicata* (Sowerby II, 1849) in Reijnen, Hoeksema & Gittenberger (2010) most likely constitutes a misidentification. Clear diagnostic characters are missing in this juvenile specimen. Based on the photographs in Reijnen, Hoeksema & Gittenberger (2010: Fig. 1E, 2F–2G) and reidentification of the specimen a similar phenotype as *Cyphoma* “black morph” (Figs. 1G and 2E) is apparent and the specimen is therefore hereafter identified as such.

### Molecular analyses

Soft tissue from the foot or mantle was used for DNA extractions. Samples were either extracted individually with the DNeasy Blood & Tissue kit, or as a part of the ‘barcoding initiative’ at Naturalis Biodiversity Center with the Machery-Nagel DNA extraction kit on a KingFisher Flex extraction robot. Extraction was performed according to the respective protocols, except for the lysis times, which were performed overnight (approx. 17 h) and the final elution volume that was decreased to 100 µL and 150 µL respectively. Before PCR amplification, extracts were diluted 100 to 300 times to lower the ratio of inhibitors vs. DNA. Each PCR reaction contained 2.5 µl CoralLoad PCR buffer, 0.5 µl dNTP’s, 1.0 µl for each primer (Table 1), 0.3 µl Taq polymerase, 18.7 µl PCR water and 1.0 µl template. For the 28S marker, 5 µl of PCR water was replaced with 5.0 µl Q-solution. Each PCR program consisted of initial denaturation for 3 min at 95 °C, followed by 39 cycles of 10 s 95 °C, specific annealing temperature (Table 1) for 1 min, with an extension of 1 min. A final extension of 10 min was used as a final step in the PCR programme. PCR amplification was performed on a C1000 Touch Thermal Cycler (Bio-RAD). Sequencing of the PCR products was performed at either Macrogen Europe (Amsterdam, The Netherlands) or at BaseClear (Leiden, The Netherlands) on an ABI Automated Sequencer 3730xl capillary sequencer. Sequences were edited in Sequencher 4.10.1. All novel sequences were uploaded to GenBank (accession numbers: KT372440 –KT372515 and KX360169 –KX360219). Additional sequences of Caribbean ovulids (Reijnen, Hoeksema & Gittenberger, 2010) were downloaded from GenBank (Table S1) and aligned on the GUIDANCE server (Penn et al., 2010) using the MAFFT algorithm (alignment score: 0.792612). Gene regions that could not be amplified for certain specimens were replaced by “N” in the final alignment. DNA amplification of a specimen of *Cyphoma* cf. *alleneae* (Fig. 1D), collected from Curaçao in 2005, was unsuccessful.

**Table 1:** Primer information of the markers used in this study, including annealing temperatures, sequenced regions and fragment sizes.

Primer names	Primer sequence	Region	Annealing T	Fragment size (bp)	Reference
H3F	ATGGCTCGTACCAAGCAGACVGC	Histone H3 (nuclear)	50	∼380	Colgan, Ponder & Eggler (2000)
H3R	ATATCCTTRGGCATRATRGTGAC	Histone H3 (nuclear)	50	∼380	Colgan, Ponder & Eggler (2000)
LSU5	TAGGTCGACCCGCTGAAYTTAAGCA	28S (nuclear)	50	∼800	Littlewood, Curini-Galletti & Herniou (2000)
LSU800rc	GACTCCTTGGTCCGTGTTTC	28S (nuclear)	50	∼800	This publication
16Sar	CGCCTGTTTATCAAAAACAT	16S (mitochondrial)	52	∼540	Palumbi (1996)
16Sbr	CCGGTCTGAACTCAGATCACGT	16S (mitochondrial)	52	∼540	Palumbi (1996)
LCO-1490	GGTCAACAAATCATAAAGATATTGG	COI (mitochondrial)	50	∼660	Folmer et al. (1994)
HCO-2198	TAAACTTCAGGGTGACCAAAAATCA	COI (mitochondrial)	50	∼660	Folmer et al. (1994)

The final alignment contained 46 specimens (Table 2; Table S1) and the concatenated dataset was 2,355 base pairs in length including insertions and/or deletions. The Indo-Pacific species *Ovula ovum* (Linnaeus, 1758) was selected as outgroup. The datasets of the individual markers were subjected to the model-testing algorithm in jModeltest (Darriba et al., 2012) and MEGA6 (Tamura et al., 2013) based on the uncorrected Akaike Information Criterion (16S: GTR + G; 28S: GTR + G; COI: GTR + I; H3: GTR + I). Bayesian analyses were performed in MrBayes 3.2.0 (Ronquist & Huelsenbeck, 2003) and were run for 4,000,000 generations with six chains. Trees were sampled every 100 generations. The final split frequency between the two independent runs was <0.01. Garli2.0 (Zwickl, 2006) was used to determine the phylogenetic relationships based on the maximum likelihood approach. Nodal support was assessed using 1,000 bootstrap iterations.

Additionally, gene trees were made for the four individual marker datasets. For each marker the model of evolution determined for the concatenated dataset was used. Bayesian inference analyses were performed in MrBayes, with the same settings as for the concatenated dataset. The final split frequency between the two independent runs was <0.01 for all four makers.

The genetic distance between the *Cyphoma* species was assessed with the Automatic Barcode Gap Discovery tool (ABGD; Puillandre et al., 2012). Default settings were used and analysis was performed with the Jukes-Cantor (JC69) algorithm. A species delimitation assessment was performed with the species delimitation tool implemented in Geneious R8 (http://www.geneious.com, Kearse et al., 2012). A Bayesian phylogeny based on four million iterations was used for the species delimitation analysis. Genetic distances were calculated in MEGA6 and minimum spanning haplotype networks for COI and 16S were constructed in PopART (http://popart.otago.ac.nz). Figure 3 shows the different haplotypes per locality for 16S and COI.

**Table 2:** Genetic variation (%) in COI of Atlantic Ovulidae between and within *Cyphoma* species groups.

Between groups (no. of specimens)	1	2	3	4	6	7	8	9	Within groups	
1. *Cyphoma gibbosum* (*n* = 18)									*Cyphoma gibbosum*	0.1
2. *Cyphoma* sp. (*n* = 3)	0.1								*Cyphoma* sp.	0.1
3. *Cyphoma signatum* (*n* = 6)	0.2	0.2							*Cyphoma signatum*	0.2
4. *Cyphoma*. *mcgintyi* (*n* = 3)	0.3	0.4	0.3						*Cyphoma mcgintyi*	0.2
5. *Simnialena uniplicata* (*n* = 1)	0.3	0.4	0.2	0.6					*Simnialena uniplicata*	–
6. *Cymbovula acicularis* (*n* = 12)	6.2	6.6	6.7	7.1	7.8				*Cymbovula acicularis*	0.2
7. *Neosimnia spelta* (*n* = 1)	6.5	7.1	6.7	7.7	7.3	7.4			*Neosimnia spelta*	–
8. *Simnia patula* (*n* = 1)	7.7	8.7	8.1	9.5	8.9	9.2	7.6		*Simnia patula*	–
9. Outgroup (*n* = 1)	9.3	9.6	10.3	10.1	11.6	10.2	12.0	12.7	Outgroup	–

**Figure 4:**
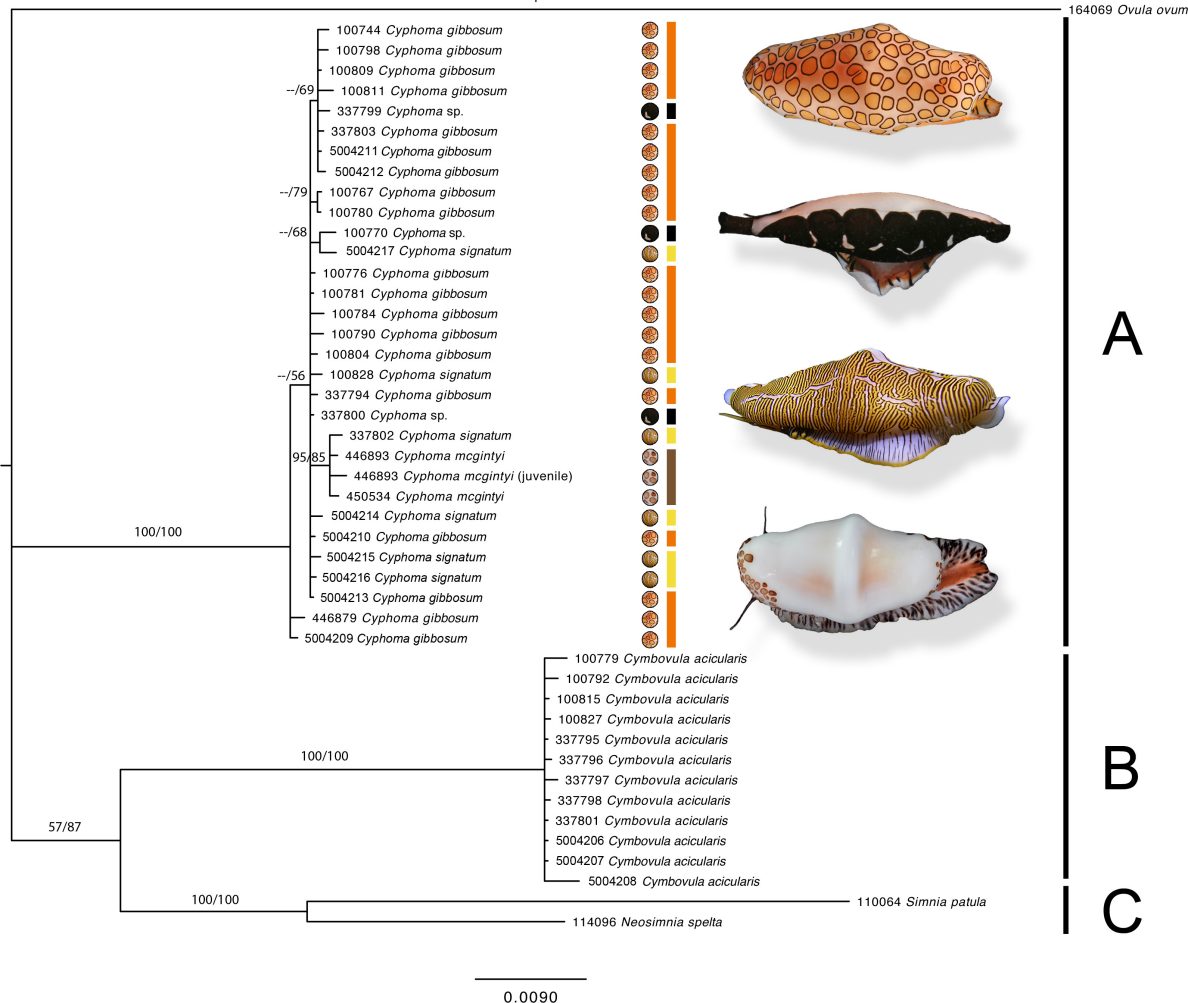
Phylogram based on the concatenated dataset of four markers. The support values in this Bayesian consensus tree are noted as Bayesian inference/Maximum likelihood. Clades A, B, and C are discussed in the results. For the *Cyphoma* species their respective characteristic mantle patterns are depicted per specimen including photographs of the live animals (not to scale). Bar colours: orange, *C. gibbosum*; yellow, *C. signatum*; brown, *C. mcgintyi*; black, *Cyphoma* “black morph”. Numbers preceding species names refer to the specimen codes in Table S1.

## Results

The phylogram (Fig. 4) based on the concatenated four gene dataset shows three groups containing: (A) *Cyphoma* spp. including *Cyphoma* “black morph” (RMNH.Mol.100770), which was formerly identified as *Simnialena uniplicata* (Reijnen, Hoeksema & Gittenberger, 2010), (B) *Cymbovula acicularis*, (C) *Neosimnia spelta* and *Simnia patula*. All groups are well supported by the Bayesian and maximum-likelihood analyses. Phylogenetic relationships between *Cymbovula acicularis* and the group containing *Neosimnia spelta* and *Simnia patula* have low support values (57/87). Within the clade containing the valid *Cyphoma* species there is no clustering observed concordant with the respective species identifications (*C. gibbosum*, *C. signatum*, *C. mcgintyi*, *Cyphoma* “black morph”). There is however a small cluster of specimens that is highly supported (95/85), which contains the three *C. mcgintyi* specimens and one representative of *C. signatum*, but the branch lengths are short. In the alignment only five nucleotide sites out of 2,355 positions support the grouping of these four specimens. One of these sites is within the non-coding 16S region, while the other four are situated in the coding COI region. Each of these sites are third codon positions, and do not change the translation of the protein coding alignment when compared with the other *Cyphoma* spp. All other *Cyphoma* species are distributed randomly throughout the clade and do not show phylogenetic affinities based on mantle patterns and colouration.

**Figure 5:**
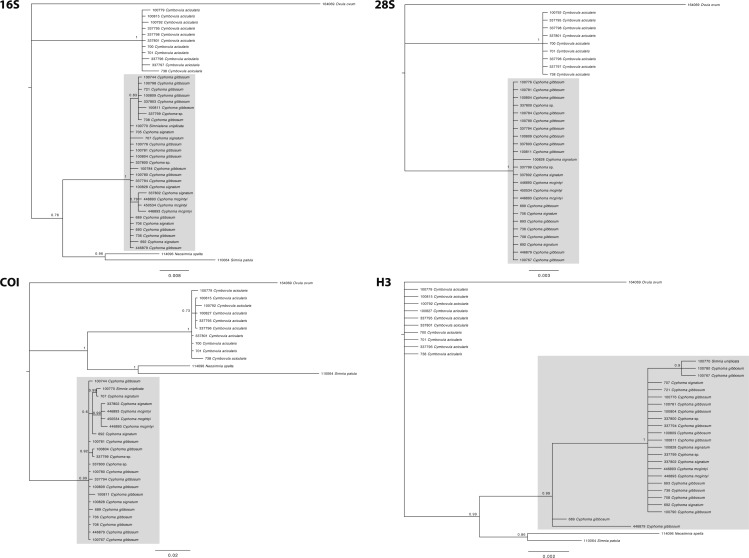
Separate gene trees. Single gene trees for the 16S, 28S, COI and H3 markers. The grey boxes highlight the clades containing the *Cyphoma* species.

The gene trees for the four independent markers (Fig. 5) show identical results to the phylogram in Fig. 4. No clustering is observed among the valid *Cyphoma* species in 16S, COI, 28S, and marginal clustering is observed in Histone H3. In the latter the clustering is based on a single base pair and/or polymorphic site and not correlated with the valid species. To investigate the observed random positioning of the *Cyphoma* species in more detail, the genetic distances between and within the species were calculated (Table 2). Genetic distance values within species (0.1–0.2%) were almost as low as between species (0.1–0.4%). When distance values were calculated between *Cyphoma* spp. and *Cymbovula acicularis*, *Simnia patula* or *Neosimnia spelta* genetic distance values were notably higher (0.1–0.4% between *Cyphoma* spp. vs. 6.5–8.1% between *Cyphoma* spp. and *Cymbovula acicularis*, *S. patula* or *N. spelta*).

The ABGD analysis resulted in five groupings: (1) *Cyphoma gibbosum*, *C. mcgintyi*, *C. signatum*, *Cyphoma* “black morph”, (2) *Cymbovula acicularis*, (3) *Neosimnia spelta*, (4) *Simnia patula*, and (5) *Ovula ovum*. The ABGD results are congruent with the results from the phylogenetic analyses and do not separate the valid *Cyphoma* species (with their unique mantle patterns and colouration) in separate groups. To test the validity of the three *Cyphoma* species and the “black morph”, a species delimitation test was performed which showed that the three species and the “black morph” should be considered a single species (P ID_strict_ < 0.95; P ID_liberal_ < 0.95). All other non-*Cyphoma* spp. were considered valid by the species delimitation test (P ID_liberal_ > 0.95).

**Figure 6:**
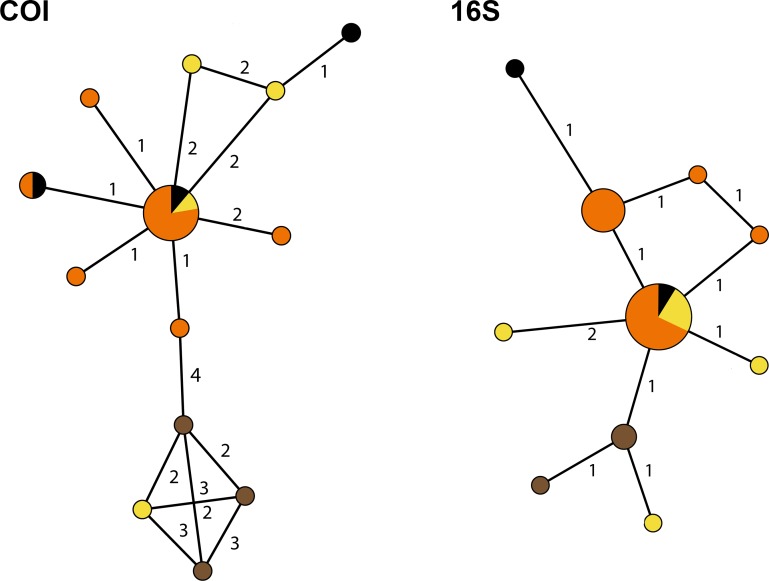
Haplotype networks for the *Cyphoma* spp. Haplotype networks for 16S and COI based on the *Cyphoma* sequence data. Orange, *C. gibbosum*; yellow, *C. signatum*; brown, *C. mcgintyi*; black, *Cyphoma* “black morph”. Numbers indicate the differences in number of base pairs.

To infer the genealogical relationships among *Cyphoma* populations, haplotype networks were created for the 16S and COI markers (Fig. 6) (there was not enough variation in the 28S and H3 nDNA sequences to create a haplotype network). The COI dataset has 17 segregating sites vs. nine in the 16S dataset. Tajima’s D statistics for both gene partitions approach zero (7.81 × 10^−8^ and 1.71 × 10^−9^ respectively). In the COI haplotype network the three *C. mcgintyi* specimens from Florida cluster together with a *C. signatum* specimen from Curaçao, with a difference of four base pairs. In the 16S haplotype network this grouping is retrieved with a single base pair difference.

## Discussion

Mantle patterns and colouration in Ovulidae were long thought to be diagnostic species characters and were used as such by several authors (Cate, 1973; Reijnen, Hoeksema & Gittenberger, 2010; Lorenz & Fehse, 2012; Lorenz & Brown, 2015). Mase (1989) did not only look at the shell and mantle, but also patterns and colours on the foot, antenna and siphon of Japanese ovulids. In this study we show that morphological characters (mantle patterns and colouration, shell morphological features) of selected species in the genus *Cyphoma* do not correspond with the genetic data, which is in accordance with studies on anatomical features in *Cyphoma* (Ghiselin & Wislon, 1966; Simone, 2004). The phylogram (Fig. 4) shows that the three *Cyphoma* species used in this study (and an unidentified morph) are indistinguishable based on two of the four molecular markers, which is also reflected in the gene trees of the independent markers (Fig. 5). Histone H3 is most informative for genus level identifications (Dinapoli et al., 2007), which could explain the clustering observed in this gene tree. The 28S gene tree clearly separates the genera *Cyphoma* and *Cymbovula*.

The COI genetic distance values between the *Cyphoma* species (Table 2) are comparable to those found in Indo-Pacific *Crenavolva* species (Reijnen, 2015). In that specific case *Crenavolva chiapponii* was synonymised with *C. aureola* based on genetic data and morphological similarity.

The haplotype networks show that the valid taxa contain only minor differences in the sequences (Fig. 6) and that the haplotypes are not linked to specific localities (Fig. 3). The observed groupings all contain multiple species and hence support the hypothesis of a single *Cyphoma* species with different morphotypes. These different morphotypes co-occur on reefs and feed on the same host species (Table S1), which refutes the idea of reproductive isolation.

The discrepancy between the different mantle colours/patterns, shell morphological characters and the molecular results in this study are difficult to reconcile. Various scenarios can, however, explain the findings presented here. Possible hypotheses include rapid diversification, supergenes/balanced polymorphism and discontinuous variation.

In a scenario of rapid divergence, trophic specialisation is frequently a key feature that characterises sister species (Vaillant, Haffner & Cristescu, 2011). Such trophic specialisation is not known in *C. gibbosum*. *Cyphoma gibbosum* is a generalist predator that has been found associated with at least 21 different host species belonging to at least nine different genera (Reijnen, Hoeksema & Gittenberger, 2010). Morphotypes resembling *Cyphoma signatum* are uncommon on most reefs and as a result ecological data are rare for this species. Most specimens have been found on the genus *Plexaurella*, yet a juvenile resembling *C. signatum* was observed on *Gorgonia ventalina* (Fig. 1F). The lack of trophic specialisation in *C. gibbosum* suggests that this is an unlikely scenario to explain the discrepancy between the genetic data and the morphological differences in mantle pattern and colouration.

A second scenario is that phenotypic diversity in *Cyphoma gibbosum* is regulated by a supergene. A supergene consists of multiple strongly linked loci that determine phenotype, without differences in the studied molecular markers (Joron et al., 2006; Joron et al., 2011). The typical orange-spotted *Cyphoma gibbosum* would be the general phenotype and rare phenotypes, in this case the yellow fingerprint pattern in *C. signatum* and the brown-spotted pattern in *C. mcgintyi*, the less common morphs (Cook, 2005). In case of the shell morphological features it is more difficult to reconcile the data. Reijnen (2015) showed that in Ovulidae minor shell morphological characters, previously used for separating nominal species, should be considered morphological variety within a single species. The presence of different morphotypes within a species is not unique within the Ovulidae. Schiaparelli et al. (2005) recognised up to three different morphotypes in one Atlantic/Mediterranean and four Indo-Pacific species (*Neosimnia spelta* (Linnaeus, 1758), *Pellasimnia brunneiterma* (Cate, 1969), *Dentiovula dorsuosa* (Hinds, 1844), *Diminovula punctata* (Duclos, 1828) and *Habuprionovolva aenigma* (Azuma & Cate, 1971)), but could not discriminate between these morphs based on 16S molecular data. Similar to the supergene hypothesis is the balanced polymorphism scenario. In both cases multiple genes regulate the mechanism, but in balanced polymorphism two alleles are maintained in a population because having heterozygote alleles is more beneficial than homozygote alleles. The balancing selection hypothesis is an unlikely scenario for our data, because the Tajima D statistic is approaching zero. This indicates that there is no selection or above normal mutation rate in the studied genes, which is expected in case of balancing polymorphism. It has to be noted that the four studied markers have to be involved in determining the phenotype, which is not known to be the case in molluscs (Schwander, Libbrecht & Keller, 2014).

A third hypothesis is that *Cyphoma gibbosum*-morphs are incipient species in the process of diverging, which is reflected by the discontinuous variation in morphology but (not yet) in the studied genes. This hypothesis is supported by the idea that phenotype precedes genotype is a common mode of speciation (Palmer, 2004). A similar case was observed in the shrimp *Conchodytes meleagrinae* (Fransen & Reijnen, 2013). Shrimp specimens from different bivalve hosts showed very dissimilar colour patterns and were thought to be distinct species. Molecular analyses showed that based on their genetic barcodes these species could not be distinguished from each other and it was therefore hypothesised that this species is in the process of speciation. Laboratory experiments, including breeding and crossing of taxa, and additional molecular approaches such as AFLPs, SNPs, microsatellites and RAD tag sequencing could be used to test the proposed hypotheses.

It is likely that shell morphological features in Ovulidae are probably more plastic than previously thought (Fig. 2; Schiaparelli et al., 2005; Reijnen, 2015) as well as in other molluscs groups (e.g., Pediculariidae (Sasaki, 2008; Braga-Henriques et al., 2011)). Strikingly, the cowrie family Cypraeidae shows contrasting outcomes from genetic analyses and multiple cryptic lineages have been discovered (Meyer, 2003; Moretzsohn, 2014). The discovery of cryptic lineages revealed using molecular data has become commonplace, but reports of distinct morphospecies attributed to a single, genetically homogeneous species are far less common (e.g., polychaetes (Willette et al., 2015, and references therein), sea stars (Harley et al., 2006), land snails (Stankowski, 2011) and caridean shrimps (Bauer, 2004)). In some of these studies no cryptic species were uncovered, but in contrast, species had to be synonymised. It is very likely that more ovulid species should be placed in synonymy, rather than described as new species.

### Taxonomic Account

Resulting from the molecular outcomes and species delimitation test, which are in line with anatomical studies by Ghiselin & Wislon (1966) and Simone (2004), *Cyphoma signatum* and *C. mcgintyi* should be synonymised with *Cyphoma gibbosum*. The synonymy of this species is therefore as follows:

**Table:** 

Family Ovulidae Fleming, 1822
Genus *Cyphoma*Röding, 1798
*Cyphoma gibbosum* (Linnaeus, 1758)


**Table:** 

*Bulla gibbosa* Linnaeus, 1758: 726
*Cyphoma dorsatum*Röding, 1798: 21
*Ovula pharetra* G. Perry, 1811: pl. 53, Fig. 2
*Ovula rostrata* Mörch, 1877: 53
*Cyphoma precursor*Dall, 1897
*Cyphoma signata* Pilsbry & McGintyi, 1939: 3, pl. 1, Figs. 1, 1A, 2, 2A, 9, 10
*Cyphoma mcgintyi* Pilsbry, 1939: 108
*Cyphoma robustior* Bayer, 1941
*Cyphoma alleneae*Cate, 1973: 67–68, Figs. 151, 151C
*?Cyphoma macumba* Petuch, 1979: 515–517, Figs. 1C–1D, 2B–2C
*Cyphoma finkli* Petuch, 1979
*Cyphoma lindae* Petuch, 1987
*Simnialena uniplicata*—Reijnen, Hoeksema & Gittenberger, 2010: Figs. 1E, 2F–2G


Remarks: Ghiselin & Wislon (1966) previously mentioned that there are no striking morphological differences between *C. gibbosum* and *C. signatum* when it comes to their functional anatomy and mantle cavity. The radular morphology of *C. gibbosum* and other Atlantic ovulids was studied by Bandel (1984) and Simone (2004) and both concluded that radular morphology does not differ significantly between ovulid species. Reid (2000) warns about using radular morphology as a morphological character, because of ecophenotypic plasticity, convergence and intraspecific variation. This study shows that in the genus *Cyphoma*, mantle patterns and colouration should also be used with care to discriminate between species, especially when the mantle is the sole differentiating character.

In contrast to Simone (2004: p.88), Lorenz & Fehse (2009) did not include *C. alleneae* in the synonymy of *C. gibbosum*, albeit without further discussion to substantiate their decision. Since there is no morphological or genetic evidence, to our knowledge, we do not consider *C. alleneae* a valid species and include this taxon in the synonymy of *C. gibbosum.*

Additionally, Simone (2004) discussed the taxonomy and systematics of other *Cyphoma* species such as *C. intermedium*, *C. macumba* and *C. signatum*. According to Simone (2004), *C. macumba* is a possible synonym of *C. signatum*. Simone (2004) investigated the type species of *C. macumba* and did not observe clear morphological differences based on the shells alone. Nevertheless, Lorenz & Fehse (2009) consider *C. macumba* and *C. signatum* separate species based on their mantle features and a minor shell morphological feature (callus-denticles on the outer labrum). Here we provisionally follow Simone’s (2004) suggestion that *C. macumba* is a synonym of *C. signatum*, and hence of *C. gibbosum*. Cate (1973) includes the following synonymies of *C. gibbosum*: *Cyphoma dorsatum* Röding, 1798, *Ovula pharetra* G. Perry, 1811, *Ovula rostrata* Mörch, 1877, and *Cyphoma precursor* Dall, 1897. Lorenz & Fehse (2009) included *C. finkli* Petuch, 1986 as a synonym of *C. signatum*, and *C. robustior* Bayer, 1941 and *C. lindae* Petuch, 1987 as synonyms of *C. mcgintyi*. We include these synonyms here as well.

Variability of morphological characters, in combination with molecular data, should be taken into account in future research on Ovulidae. Unnecessary profusion of species names and other taxonomical problems can be avoided by assessing both morphological and molecular data.

##  Supplemental Information

10.7717/peerj.3018/supp-1 Table S1 Supp. Mat. Species information and GenBank accession numbers including references. An asterisk marks the GenBank accession codes of three specimens which were misidentified in Reijnen, Hoeksema & Gittenberger (2010).

## Additional Information and Declarations

### Field Study Permissions

The following information was supplied relating to field study approvals (i.e., approving body and any reference numbers): Research on Curaçao was performed under the annual research permit (48584) issued by the Curaçaoan Ministry of Health, Environment and Nature (GMN) to the CARMABI foundation.

### Data Availability

The following information was supplied regarding data availability: Novel sequence data in GenBank (KT372440 –KT372515 and KX360169 –KX360219).
