# Supplementary material for: Coat of many colours—DNA reveals polymorphism of mantle patterns and colouration in Caribbean Cyphoma Röding, 1798 (Gastropoda, Ovulidae)
Source: PeerJ. 2017 Mar 2;5:e3018. doi: 10.7717/peerj.3018 (PMC5337085; doi:10.7717/peerj.3018)
Supplement: Table S1 — An asterisk marks the GenBank accession codes of three specimens which were misidentified in Reijnen, Hoeksema & Gittenberger (2010). [file peerj-05-3018-s001.docx]

**Table S1.** Species information and GenBank accession numbers including references. An asterisk marks the GenBank accession numbers of three specimens that were misidentified in Reijnen, Hoeksema & Gittenberger, 2010.

| Species | Code | Location, locality code | Longitude / Latitude | Depth (m) | Host coral | COI | 16S | 28S | H3 | Reference |
| --- | --- | --- | --- | --- | --- | --- | --- | --- | --- | --- |
| *Cymbovula acicularis* (Lamarck, 1810) | RMNH.Mol.100779 | Curaçao, Barank'i Karanito, CUR.15 | 12°02'13.5"N 68°48'14.2"W | 25 | *Antillogorgia acerosa* (Pallas, 1766) | GU363447 * | GU363434 | - | KT372494 | Reijnen, Hoeksema & Gittenberger, 2010; This publication |
| *Cymbovula acicularis* (Lamarck, 1810) | RMNH.Mol.100792 | Curaçao, Daaibooi, CUR.19 | 12°12'42.9"N 69°05'0°5.4"W | 6 | *Gorgonia ventalina* (Linnaeus, 1758) | GU363448 | GU363436 | KT372474 | KT372497 | Reijnen, Hoeksema & Gittenberger, 2010; This publication |
| *Cymbovula acicularis* (Lamarck, 1810) | RMNH.Mol.100815 | Curaçao, Caracasbaai, CUR.17 | 12°04'25.8"N 68°51'46.5"W | 17 | *Antillogorgia bipinnata* (Verrill, 1864) | GU363449 | GU363437 | - | KT372501 | Reijnen, Hoeksema & Gittenberger, 2010; This publication |
| *Cymbovula acicularis* (Lamarck, 1810) | RMNH.Mol.100827 | Curaçao, Marie Pampoen/Carpile, CUR.05 | 12°05'42.1"N 68°54'43.0"W | 11 | *Gorgonia ventalina* (Linnaeus, 1758) | KX360174 | - | - | KX360186 | This publication |
| *Cymbovula acicularis* (Lamarck, 1810) | RMNH.Mol.337795 | Curaçao, Blauwbaai, CAO.04 | 12°08'05.7"N 68°59'03.5"W | 10 | *Gorgonia ventalina* (Linnaeus, 1758) | KT372445 | KT372460 | KT372481 | KT372507 | This publication |
| *Cymbovula acicularis* (Lamarck, 1810) | RMNH.Mol.337796 | Curaçao, St. Marie, CAO.06 | 12°11'53.0"N 69°04'45.1"W | 14 | *Antillogorgia acerosa* (Pallas, 1766) | KT372446 | KT372461 | KT372482 | KT372508 | This publication |
| *Cymbovula acicularis* (Lamarck, 1810) | RMNH.Mol.337797 | Curaçao, Playa Jeremy, CAO.07 | 12°19'44.5"N 69°09'00.5"W | 9 | *Antillogorgia* sp. | - | KX360219 | KX360201 | - | This publication |
| *Cymbovula acicularis* (Lamarck, 1810) | RMNH.Mol.337798 | Curaçao, Waterfabriek II, CAO.10 | 12°06'37.0"N 68°57'16.5"W | 4 | *Gorgonia ventalina* (Linnaeus, 1758) | - | KX360215 | KX360198 | - | This publication |
| *Cymbovula acicularis* (Lamarck, 1810) | RMNH.Mol.337801 | Curaçao, Marie Pampoen, CAO.21 | 12°05'26.7"N 68°54'17.8"W | 10 | *Antillogorgia acerosa* (Pallas, 1766) | KT372449 | KT372464 | KT372485 | KT372511 | This publication |
| *Cymbovula acicularis* (Lamarck, 1810) | RMNH.5004206 | St. Eustatius, The Blocks, EUX015 | 17°27'50.9"N 62°59'06.8"W | - | - | KX360175 | KX360216 | KX360199 | KX360189 | This publication |
| *Cymbovula acicularis* (Lamarck, 1810) | RMNH.5004207 | St. Eustatius, The Blocks, EUX015 | 17°27'50.9"N 62°59'06.8"W | - | *-* | KX360176 | KX360217 | KX360200 | KX360188 | This publication |
| *Cymbovula acicularis* (Lamarck, 1810) | RMNH.5004208 | St. Eustatius, Blind Shoal, EUX039 | 17°30'37.1"N 63°00'27.1"W | - | *Antillogorgia* sp. | KX360177 | KX360218 | KX360202 | KX360189 | This publication |
| *Cyphoma gibbosum* (Linnaeus, 1758) | RMNH.Mol.100767 | Curaçao, Blauwbaai, CUR.10 | 12°22'29.9"N 68°59'27.7"W | 8 | *Antillogorgia acerosa* (Pallas, 1766) | KT372440 | - | KT372470 | KT372491 | This publication |
| *Cyphoma gibbosum* (Linnaeus, 1758) | RMNH.Mol.100776 | Curaçao, Playa Jeremy, CUR.12 | 12°19'44.1"N 69°09'00.2"W | 17 | *Briareum asbestinum* (Pallas, 1766) | - | KT372455 | KT372471 | KT372493 | This publication |
| *Cyphoma gibbosum* (Linnaeus, 1758) | RMNH.Mol.100780 | Curaçao, Barank'i Karanito, CUR.15 | 12°02'13.5"N 68°48'14.2"W | 10 | *Gorgonia ventalina* (Linnaeus, 1758) | GU363440 | GU363428 | KT372472 | KT372495 | Reijnen, Hoeksema & Gittenberger, 2010; This publication |
| *Cyphoma gibbosum* (Linnaeus, 1758) | RMNH.Mol.100781 | Curaçao, Barank'i Karanito, CUR.15 | 12°02'13.5"N 68°48'14.2"W | 9 | *Plexaurella nutans* (Duchassaing & Michelotti, 1860) | GU363444 | GU363432 | KT372473 | KT372496 | Reijnen, Hoeksema & Gittenberger, 2010; This publication |
| *Cyphoma gibbosum* (Linnaeus, 1758) | RMNH.Mol.100784 | Curaçao, Atlantis diving/Drielstraat, CUR.02 | 12°05'42.1"N 68°54'43.0"W | 21 | *Antillogorgia bipinnata* (Verrill, 1864) | - | KX360207 | KX360190 | - | This publication |
| *Cyphoma gibbosum* (Linnaeus, 1758) | RMNH.Mol.100790 | Curaçao, Santa Martha, CUR.18 | 12°16'04.9"N 69°07'43.6"W | 17 | *Plexaurella dichotoma* (Esper, 1791) | - | - | - | KX360185 | This publication |
| *Cyphoma gibbosum* (Linnaeus, 1758) | RMNH.Mol.100798 | Curaçao, Santa Martha, CUR.18 | 12°16'04.9"N 69°07'43.6"W | 5 | *Pterogorgia citrina* (Esper, 1792) | - | KX360203 | - | - | This publication |
| *Cyphoma gibbosum* (Linnaeus, 1758) | RMNH.Mol.100804 | Curaçao, Sint Michielsbaai, CUR.21 | 12°08'50.9"N 68°59'56.6"W | 14 | *Pseudoplexaura porosa* (Houttuyn, 1772) | GU363443 | GU363431 | KT372475 | KT372498 | Reijnen, Hoeksema & Gittenberger, 2010; This publication |
| *Cyphoma gibbosum* (Linnaeus, 1758) | RMNH.Mol.100809 | Curaçao, Superior Producer, CUR.22 | 12°05'21.5"N 68°56'35.5"W | 18 | *Antillogorgia americana* (Gmelin, 1791) | GU363446 * | GU363433 | KT372476 | KT372499 | Reijnen, Hoeksema & Gittenberger, 2010; This publication |
| *Cyphoma gibbosum* (Linnaeus, 1758) | RMNH.Mol.100811 | Curaçao, Superior Producer, CUR.22 | 12°05'21.5"N 68°56'35.5"W | 5 | *Muricea muricata* (Pallas, 1766) | GU363441 | GU363429 | KT372477 | KT372500 | Reijnen, Hoeksema & Gittenberger, 2010; This publication |
| *Cyphoma gibbosum* (Linnaeus, 1758) | RMNH.Mol.337794 | Curaçao, Waterfabriek I, CAO.02 | 12°06'31.0"N 68°57'01.2"W | - | *Eunicea* sp. | KT337794 | KT372459 | KT372480 | KT372506 | This publication |
| *Cyphoma gibbosum* (Linnaeus, 1758) | RMNH.Mol.337803 | Curaçao, Grote Knip, CAO.22 | 12°21'04.1"N 69°09'06.9"W | 10 | *-* | - | KX360208 | KX360191 | - | This publication |
| *Cyphoma gibbosum* (Linnaeus, 1758) | UF.446879 | USA, Florida, N of St. Petersburg | 28°35'55.7"N 84°15'41.4"W | 27 | unknown | KT372451 | KT372466 | KT372487 | KT372513 | This publication |
| *Cyphoma gibbosum* (Linnaeus, 1758) | RMNH.5004209 | St. Eustatius, Aquarium, EUX012 | 17°30'22.6"N 63°00'22.0"W | - | - | KX360170 | KX360209 | KX360192 | KX360180 | This publication |
| *Cyphoma gibbosum* (Linnaeus, 1758) | RMNH.5004210 | St. Eustatius, Aquarium, EUX012 | 17°30'22.6"N 63°00'22.0"W | - | - | - | KX360211 | KX360194 | KX360181 | This publication |
| *Cyphoma gibbosum* (Linnaeus, 1758) | RMNH.5004211 | St. Eustatius, Blairs Reef, EUX019 | 17°28'13.6"N 62°59'30.2"W | - | *Plexaura nina* Bayer & Deichmann, 1958 / *homomalla* (Esper, 1792) | KX360172 | KX360213 | KX360196 | KX360183 | This publication |
| *Cyphoma gibbosum* (Linnaeus, 1758) | RMNH.5004212 | St. Eustatius, Twelve Guns, EUX029 | 17°28'12.8"N 62°58'58.7"W | - | - | - | KX360206 | - | KX360179 | This publication |
| *Cyphoma gibbosum* (Linnaeus, 1758) | RMNH.5004213 | St. Eustatius, Blue Bead Hole II, EUX037 | 17°28'37.4"N 62°59'29.6"W | - | - | KX360171 | KX360212 | KX360195 | KX5004213 | This publication |
| *Cyphoma mcgintyi* (Pilsbry, 1939) | UF.446893a | USA, Florida, N of St. Petersburg | 28°32'16.1"N 84°16'21.7"W | 26 | unknown | KT372452 | KT372467 | KT372488 | KT372514 | This publication |
| *Cyphoma mcgintyi* (Pilsbry, 1939) | UF.446893b | USA, Florida, N of St. Petersburg | 28°32'16.1"N 84°16'21.7"W | 26 | unknown | KT372453 | KT372468 | KT372489 | KT372515 | This publication |
| *Cyphoma mcgintyi* (Pilsbry, 1939) | UF.450534 | USA, Florida, NNW of St. Petersburg, S of Big Bend area | 28°39'04.0"N 84°23'03.8"W | 26-30 | unknown | KT372454 | KT372469 | KT372490 | - | This publication |
| *Cyphoma signatum* Pilsbry & McGinty, 1939 | RMNH.Mol.100828 | Curaçao, Marie Pampoen/Carpile, CUR.05 | 12°05'42.1"N 68°54'43.0"W | 5 | *Plexaurella dichotoma* (Esper, 1791) | KT372441 | KT372456 | KT372478 | KT372502 | This publication |
| *Cyphoma signatum* Pilsbry & McGinty, 1939 | RMNH.Mol.337802 | Curaçao, Marie Pampoen, CAO.21 | 12°05'26.7"N 68°54'17.8"W | 8 | *Gorgonia ventalina* (Linnaeus, 1758) | KT372450 | KT372465 | KT372486 | KT372512 | This publication |
| *Cyphoma signatum* Pilsbry & McGinty, 1939 | RMNH.5004214 | St. Eustatius, Aquarium, EUX012 | 17°30'22.6"N 63°00'22.0"W | - | *-* | KX360173 | KX360214 | KX360197 | KX360184 | This publication |
| *Cyphoma signatum* Pilsbry & McGinty, 1939 | RMNH.5004215 | St. Eustatius, Shark Reef, EUX018 | 17°30'37.1"N 63°00'27.1"W | - | *Plexaurella nutans* (Duchassaing & Michelotti, 1860) | - | KX360204 | - | - | This publication |
| *Cyphoma signatum* Pilsbry & McGinty, 1939 | RMNH.5004216 | St. Eustatius, Blairs Reef, EUX019 | 17°28'13.6"N 62°59'30.2"W |  | *Plexaurella dichotoma* (Esper, 1791) | - | KX360210 | KX360193 | - | This publication |
| *Cyphoma signatum* Pilsbry & McGinty, 1939 | RMNH.5004217 | St. Eustatius, Shark Reef, EUX018 | 17°30'37.1"N 63°00'27.1"W | - | *Plexaurella nutans* (Duchassaing & Michelotti, 1860) | KX360169 | KX360205 | - | KX360178 | This publication |
| *Cyphoma* sp. | RMNH.Mol.337799 | Curaçao, Holiday Beach, CAO.11 | 12°06'34.1"N 68°56'49.3"W | 4 | *Eunicea tourneforti* Milne Edwards & Haime, 1857 | KT372447 | KT372462 | KT372483 | KT372509 | This publication |
| *Cyphoma* sp. | RMNH.Mol.337800 | Curaçao, Holiday Beach, CAO.11 | 12°06'34.1"N 68°56'49.3"W | 4 | *Eunicea tourneforti* Milne Edwards & Haime, 1858 | KT372448 | KT372463 | KT372484 | KT372510 | This publication |
| *Neosimnia spelta* (Linnaeus, 1758) | RMNH.Mol.114096 | Spain, Begur, Aigua Blava | 41°56'08,0"N 03°13'04,9"E | <15 | *Leptogorgia sarmentosa* (Esper, 1789) | KT372442 | KT372457 | - | KT372504 | This publication |
| *Ovula ovum* (Linnaeus, 1758) | RMNH.Mol.164069 | Malaysia, Borneo, Kapalai Island, SEM.10 | 04°13'04,8"N 118°40'20,1"E | 3 | *Sarcophyton glaucum* (Quoy & Gaimard, 1833) | KT372443 | KT372458 | KT372479 | KT372505 | This publication |
| *Simnia patula* (Penant, 1777) | RMNH.Mol.110064 | North Sea, S side of Doggersbank | 54°20'N 02°20'E | - | *Alcyonium digitatum* Linnaeus, 1758 | GU363450 | GU363438 | - | KT372503 | Reijnen, Hoeksema & Gittenberger, 2010; This publication |
| *Simnialena uniplicata* (Sowerby II, 1848) | RMNH.Mol.100770 | Curaçao, Marie Pampoen/Carpile, CUR.05 | 12°05'42.1"N 68°54'43.0"W | 18 | *Gorgonia flabellum* Linnaeus, 1758 | GU363445 * | GU363435 | - | KT372492 | Reijnen, Hoeksema & Gittenberger, 2010; This publication |
